# Determinants of adherence to antiretroviral drugs among people living with HIV/AIDS in the Ife-Ijesa zone of Osun state, Nigeria

**DOI:** 10.4102/phcfm.v1i1.6

**Published:** 2009-04-15

**Authors:** Muhammed O. Afolabi, Kayode T. Ijadunola, Adesegun O. Fatusi, Olayinka A. Olasode

**Affiliations:** 1 Department of Family Medicine, Ladoke Akintola University Teaching Hospital, Nigeria; 2 Department of Community Health, Obafemi Awolowo University, Nigeria; 3 Department of Dermatology and Venereology, Obafemi Awolowo University, Nigeria

**Keywords:** Factors, adherence, PLWHA, antiretroviral drugs, Nigeria

## Abstract

**Background:**

The advent of antiretroviral (ARV) drugs has transformed HIV/AIDS into a chronic manageable disease and strict adherence is required for the medication to be effective. However, factors influencing adherence to ARV therapy (ART) vary from country to country.

**Method:**

120 subjects who received ARV drugs at a federal government-designated ART site located within the Obafemi Awolowo University Teaching Hospital complex, (OAUTHC), Ile-Ife, and a community-based non-governmental organisation, Living Hope Care (LIHOC), Ilesa, from February to May 2006 were serially recruited and studied. Relevant data were collected using an interviewer-administered, patient medication adherence questionnaire. Focus group discussions were also held among the subjects to further elicit qualitative information on factors influencing adherence to ART.

**Results:**

The age of participants ranged from 21 to 65 years with a mean age of 40.2 + 10.3 years. Participants had been on ARV drugs for a period ranging between three and 60 months. The overall adherence rate in the study population was 44%. 66% of participants who accessed ARV drugs from LIHOC, Ilesa, had good adherence while only 14% of participants who accessed ARV drugs from OAUTHC, Ile-Ife, had good adherence. Participants with good adherence did not pay funds for the preliminary ARV eligibility investigations and they were also offered regular adherence counselling. These facilities were barely available in the group with poor adherence. Demographic factors such as age, gender and marital status did not seem to have any significant association with adherence level (p > 0.05).

**Conclusion:**

The level of adherence was high in a cohort of PLWHA accessing ARV drugs in Ilesa while it was low among PLWHA receiving ART in Ife. The most important reasons for this difference were lack of funds for investigations and poor psycho-social counselling.

## INTRODUCTION

The development of antiretroviral (ARV) drugs has transformed HIV/AIDS to that of a chronic manageable disease. Studies have reported improved quality of life of PLWHA on ARV therapy (ART), reduced progression of the disease and declining mortality from the pandemic.^1, 2^ However, strict adherence is required to achieve therapeutic success in HIV management. Paterson et al. suggest that near-perfect adherence, that is, higher than 95%, is necessary to achieve suppression of HIV replication (HIV-RNA < 400 copies/ml).^3^ Other data, however, suggest that adherence levels of 100% achieve even greater benefits.^4, 5^ Inadequate viral suppression resulting from failure to adhere closely to treatment causes a worsening of immunological and clinical states, which may eventually lead to emergence of drug-resistant HIV strains.^6^

The consequences of low adherence are serious for the individual, public health and the optimal use of limited health care resources.^6^ Non-adherence in patients on anti-HIV therapy is the strongest predictor of failure to achieve viral suppression below the level of detection.^7^ Conscientious treatment adherence is difficult under any circumstances, considering the unforgiving nature of HIV replication, the complexity of the highly active antiretroviral therapy (HAART) regimens, and associated short- and long-term toxicity. Adherence to HAART becomes a central issue of concern, not only because factors that influence a patient's ability to adhere are multiple and complex, but also because treatment resistance can occur for an entire class of ARV drugs and ultimately renders HAART ineffective. In addition, resistant viral strain of HIV can be transmitted to newly infected individuals who will therefore have fewer effective treatment options from the start of their HIV infections.

Studies have reported determinants of adherence to ARV medication.^8–20^ While socio-demographic characteristics were reported not to be associated with adherence in some studies,^8, 9^ factors such as younger age,^10, 11^ African descent,^12, 13^ low income,^14^ low levels of education^15^ and psychological factors such as depression,^16^ high levels of stress,^9^ psychiatric conditions,^3^ excessive drinking,^17^ and drug use^18^ were associated with adherence. Some studies also suggest that non-adherence tends to increase with the number of times medications are taken per day^19^ and the number of different medications.^13^ Furthermore, patients who are experiencing adverse effects are less likely to adhere than patients who are tolerating the medication.^20^

There is a dearth of data on factors influencing adherence to ART in Nigeria, and this study therefore aims to address this gap.

## METHOD

### Study location

The study was conducted in Ile-Ife and Ilesa, two adjoining towns in Osun State in south-west Nigeria. The two towns form the nucleus of the Ife-Ijesa geo-political zone and house the two units of the Obafemi Awolowo University Teaching Hospital, Ile-Ife, which is one of the ART centres approved by the federal government of Nigeria. Although the HIV prevalence of Osun State was estimated at 1.2%, the state was considered a ‘hotspot’ for HIV transmission in view of numerous junction towns that serve as transit centres for long-distance drivers and travellers to other major cities in Nigeria.^22^ ARV drugs are available free of charge from the federal government, but beneficiaries are expected to undergo mandatory laboratory investigations before commencement of the medication. These investigations cost about 13,000 naira (about $US104), and are repeated every three months to monitor progress of the patients on the ARV drugs.

LIHOC is a non-governmental, community-based organisation that provides comprehensive care and support for PLWHA. The organisation is situated in Ilesa and collaborates with one of the US President's Emergency Plan for AIDS Relief (PEPFAR) sites located within the Nigerian Institute of Medical Research, Lagos. Clients from LIHOC access ARV drugs from the PEPFAR facility in Lagos. The laboratory investigations as well as ARV drugs are provided free for all clients, including those from LIHOC, Ilesa. PLWHA are trained in adherence counselling and home-based care. They are also organised into support groups that meet every fortnight to share experiences and discuss issues that affect them. However, at the ART site in Ile-Ife, adherence and psycho-social support were barely existent at the time of the study. The investigator attended a number of such meetings in Ile-Ife and Ilesa.

### Study design

The study employed a cross-sectional, descriptive design.

#### Study population

These were patients who tested positive for HIV and were receiving ART in the two recognised ART centres located in the Ife-Ijesa zone of Osun State. These patients had undergone rapid screening tests using HIV 1/2 STAT-PAK and confirmatory tests were done using the Western blot method. The patients underwent further investigations to rule out opportunistic infections such as pulmonary tuberculosis. In the absence of this infection, a CD4 count of ≤ 200 µl/ml is used as cut-off value for commencement of ART. At the time of study, very few HIV-positive people were accessing ARV drugs at the centres.

#### Sampling technique

Participants were serially recruited over the study period. All PLWHA on ART were eligible to participate, except those that satisfied the exclusion criteria. These criteria included PLWHA who were acutely ill or those that refused to grant consent for the study. Out of 75 clients approached in the Ilesa site, 70 agreed to participate in the study, while 50 clients consented in the Ile-Ife site out of 53 approached. There was no significant difference in age, sex and other socio-demographic factors between those selected and the rest of the clients at the two sites.

#### Data-collection instruments

Data were collected using quantitative and qualitative techniques. The quantitative instrument consisted of a semi-structured, interviewer-administered questionnaire that contains sections on socio-demographic data and factors influencing adherence to ART. Adherence to ART in the previous seven days of the interview was measured by self-report. Initially, the percentage of adherence was calculated for each drug by dividing the number of pills taken by the number of pills prescribed. Then, the percentage of adherence to the antiretroviral ART was estimated by the average of adherence to the drugs. A score of 95% and above represented good adherence and less than 95% was rated as poor adherence.^3^

To ensure further exploration of factors influencing adherence to ART among PLWHA, two focus group discussion sessions were held each in Ile-Ife (male and female groups) and Ilesa (male and female groups) using a focus group discussion guide. Participants were segregated by sex to ensure homogeneity and open discussion among same-sex clients. Six to eight clients who had not answered the questionnaires were included in each focus group discussion. A total of 28 clients were randomly selected from the register and 24 people agreed to participate. The focus group discussion sessions were audio-taped and later transcribed. The investigator served as the facilitator for the focus group discussions.

#### Data analysis

Quantitative data were analysed using SPSS for Windows, Version 11. Descriptive and inferential statistical tests were employed. These included bivariate (chi-square) and multivariate (logistic regression) analyses to determine correlates or predictors of adherence to ART. The independent predictors of adherence were assessed using a sequence of multivariate logistic regression models. A model was set up with adjustments for socio-demographic and ART variables. Variables selected for inclusion in the subsequent model were based on their significance (that is p < 0.05) in bivariate analyses or in the prior model.

Qualitative data gathered from focus group discussions were analysed through detailed content analysis and ethnographic summary. This involved verbatim quoting of participants to buttress certain arguments raised in the course of discussion.

#### Ethical clearance and consent

Ethical clearance was obtained from the Research and Ethical Committee of OAUTHC, Ile-Ife. Informed written consent was obtained from each participant before the interview.

## RESULTS

One hundred and twenty (120) PLWHA receiving ART at OAUTHC, Ile-Ife, and LIHOC, Ilesa, were studied. 50 subjects (41.7%) were recruited from OAUTHC, Ile-Ife, while 70 subjects (58.3%) were recruited from LIHOC, Ilesa. The ages of the participants ranged from 21 to 65 years; the mean age was 40.2 ± 10.3 years. Participants had been on ART for a period ranging between three and 60 months and the mean period of commencement of ART was 16.9 ± 12.3 months.

Table 1 shows that the majority of the clients from the two sites were female, about half were currently married, and that a higher proportion (64.3%) of participants attended support group meetings regularly in Ilesa than those in Ile-Ife.

**TABLE 1:** Socio-demographic distribution of participants

CHARACTERISTICS	ILE-IFE (n = 50)	ILESA (n = 70)
**AGE GROUP (YEARS)**		
15–24	0 (0.0)	5 (7.1)
25–34	9 (18.0)	22 (31.4)
35–44	19 (38.0)	30 (42.9)
≥ 45	22 (44.0)	13 (18.6)
**SEX**		
Female	31 (62.0)	48 (68.6)
Male	19 (38.0)	22 (31.4)
**MARITAL STATUS**		
Single	0 (0.0)	14 (20.0)
Married	28 (56.0)	35 (50.0)
Separated	0 (0.0)	3 (4.3)
Divorced	2 (4.0)	2 (2.9)
Widowed	19 (38.0)	16 (22.9)
**LEVEL OF EDUCATION**		
No formal education	24 (48.0)	21 (30.0)
Primary	11 (22.0)	12 (17.1)
Secondary	10 (20.0)	23 (32.9)
Tertiary	15 (30.0)	14 (20.0)
**RELIGION**		
Christianity	44 (88.0)	55 (78.6)
Islam	6 (12.0)	15 (21.4)
**SUPPORT GROUP ATTENDANCE**		
Regularly	20 (40.0)	45 (64.3)
Not regularly	30 (60.0)	25 (35.7)

**FIGURE 1:**
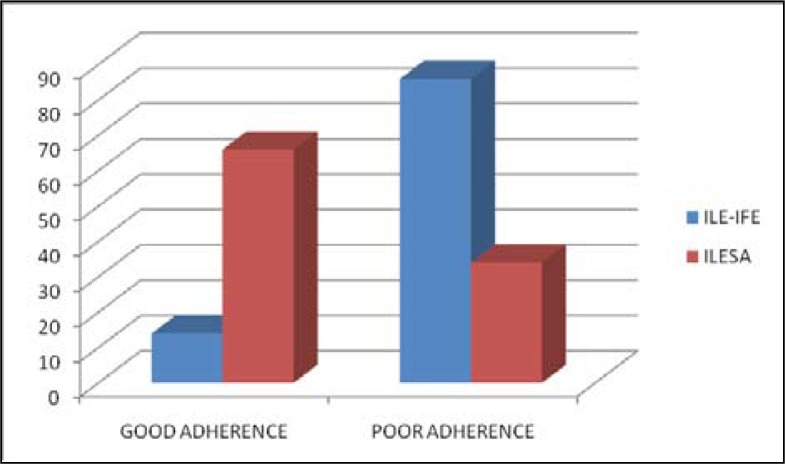
Bar chart showing level of ART adherence among study participants

Figure 1 shows that 14% of subjects that accessed ARV drugs from Ile-Ife had good adherence compared to 66% of those that accessed care from Ilesa.

Table 2 shows that participants with formal education were significantly more likely to adhere to ART (63%) than were those with no formal education (37%), (p < 0.0001). Similarly, other significant correlates of adherence included total monthly income of participants, their affordability of the cost of ARV drugs, their sources of ARV drugs and their membership/attendance at support groups (p < 0.01). Further correlates included the occupational status of the participants and their perceptions of ARV dosage regimens (p < 0.01).

**TABLE 2:** Distribution of demographic and socio-economic characteristics of study participants by their ART adherence pattern

CHARACTERISTICS	GOOD ADHERENCE	POOR ADHERENCE	SIGNIFICANCE
**EDUCATION STATUS**			*χ^2^* = 27.758
No formal education	6 (13.3)	39 (86.7)	df = 1
Formal education	47 (62.7)	28 (37.3)	p = **0.000**
**TOTAL MONTHLY INCOME**			*χ^2^* = 23.024
Less than N5 000	22 (28.2)	56 (71.8)	df = 1
N5 000 and more	31 (73.8)	11 (26.2)	p = **0.000**
**SOURCES OF ARV DRUGS**			*χ^2^* = 31.631
Ife	7 (14.0%)	43 (86.0%)	df = 1
Ilesa	46 (65.7%)	24 (34.3%)	p = **0.000**
**AFFORDABILITY OF ARV DRUGS**			*χ^2^* = 11.007
Can easily afford costs	20 (71.4%)	8 (28.6%)	df = 1
Find cost of ARV drugs expensive	33 (35.9%)	59 (64.1%)	p = **0.001**
**SUPPORT GROUP ATTENDANCE**			*χ^2^* = 9.358
Yes	37 (56.9%)	28 (43.1%)	df = 1
No	16 (29.1%)	39 (70.9%)	p = **0.002**
**OCCUPATION**			*χ^2^* = 7.019
Currently employed	33 (37.1)	56 (62.9)	df = 1
Not currently employed	20 (64.5)	11 (35.5)	p = **0.008**
**PERCEPTIONS OF ARV DOSAGE REGIMEN**			*χ^2^* = 5.680
Find frequency cumbersome	2 (20.0%)	8 (80.0%)	df = 1
Do not find it cumbersome	45 (40.9%)	65 (59.1%)	p = **0.017**
**AGE GROUP**			*χ^2^* = 0.348
Less than 45 years	39 (45.9)	46 (54.1)	df = 1
45 years and older	14 (40.0)	21 (60.0)	p = 0.555
**SEX**			*χ^2^* = 0.538
Female	33 (41.8%)	46 (58.2%)	df = 1
Male	20 (48.8%)	21 (51.2%)	p = 0.463
**MARITAL STATUS**			*χ^2^* = 0.187
Currently married	29 (46.0)	34 (54.0)	df =1
Not currently married	24 (42.1)	33 (57.9)	p = 0.665
**PERCEPTIONS OF ATTITUDES OF SERVICE PROVIDERS**			*χ^2^* =0.541
It is a barrier to adherence	44 (45.8%)	52 (54.2%)	df= 1
It is not a barrier to adherence	9 (37.5%)	15 (62.5%)	p = 0.462
**PERCEPTIONS OF SIDE EFFECTS OF ARV DRUGS**			*χ^2^* = 2.307
It is a barrier to adherence	5 (27.8%)	13 (72.2%)	df = 1
It is not a barrier to adherence	48 (47.1%)	54 (52.9%)	p = 0.129

Table 3 shows that the true predictors of adherence to ART among PLWHA in the Ife-Ijesa zone were sources of ART, educational attainment and total monthly income. Participants who attended and got their ARV drugs from LIHOC, Ilesa, were more likely to adhere to ART than were those who attended OAUTHC, Ile-Ife. Similarly, participants who earned a total monthly income of N5 000 (about US$40) or more and those who had formal education were more likely to adhere than were those who earned less than N5 000 and those without formal education.

**TABLE 3:** Logistic regression analyses of the significant correlates of adherence to ART among study participants

	B	S.E.	df	Sig.	95.0% C.I. for EXP(B)	

					LOWER	UPPER
**Sources of ARV drugs**	2.139	0.695	1	**0.002**	2.173	33.152
**Educational attainment**	2.933	0.690	1	**0.000**	4.862	72.575
**Total monthly income**	1.308	0.627	1	**0.037**	1.082	12.656
**Affordability of ARV drugs**	-0.507	0.690	1	0.463	0.156	2.332
**Support group attendance**	-0.798	0.572	1	0.163	0.147	1.381
**Employment status**	1.182	0.683	1	0.084	0.855	12.435
**Freq. of medication**	-2.652	1.004	1	0.008	0.010	0.504

## RESULTS

A majority of the focus group discussion participants reported having missed a dose of ARV drugs. This implies that ART was not strictly adhered to. A lady from Ile-Ife remarked as follows:

‘*Although taking ARV drugs has become part of our life, we still at times forget to take it due to some reasons. I have missed my drugs sometimes. It is one of those things. One cannot be 100% compliant.’*

A male participant from Ilesa submitted that:

‘*One cannot avoid missing a dose at times. As long as the drug is available, one will take it as soon as one remembers.’*

There was, however, no report of outright discontinuation of the treatment regimen among the PLWHA that participated in this study.

Adherence to ART demands certain food requirements. Some of the participants disclosed that they were not economically buoyant to meet these food requirements. A lady from Ilesa submitted the following:

‘*Most of the time, most of us eat what is available and affordable, not necessarily what is required. To claim that we are meeting the food requirements is an understatement. Even, those without HIV are also not finding this bad economy easy.’*

Responses from the focus group discussion sessions provided further insight into the factors that influenced clients’ adherence. A majority of participants in Ile-Ife complained about the cost of investigations before commencing ART, while those in Ilesa acknowledged that ARV drugs were free of charge, but that they found it difficult to get the transport fare to Lagos where they received the drugs. One of them said:

*‘The major problem I have with ARV is the cost. Although government has made it free, we still need to obtain the drug from Lagos. The transport money then becomes a burden.’*

Another participant disclosed that:

‘*It is not that the drug is very expensive, after all, it was subsidised before, and now [it is] almost free. The major problem is that many of us are not gainfully employed. Like me, I was sacked when my employer knew I was HIV positive. This lack of regular income makes getting the drug a serious problem.’*

Very few PLWHA complained about swallowing the drug. It was also reported that the smell of the drug as well as frequency of medication did not pose any serious barrier to adherence to ART. The support received by the PLWHA was also examined. Most of them remarked that they were neither supported financially nor morally by their close relatives. A lady from Ilesa said:

*‘My friends and relatives think I can inflict HIV on them. Some of them see me as a living corpse. I was thrown out of my husband's house after his death. I received no support, except from this place.’*

Another lady from Ile-Ife lamented:

*‘My relations and friends left me alone. Even in my church the news of my HIV status called for a meeting because I was one of the church workers. I was asked to resign. They said that the news of my HIV status will negatively affect the congregation.’*

A few participants said that the adverse effects of the drugs discouraged them from taking ARV medication. A female participant from Ilesa said:

*‘When I was having rashes, I reduced ARV intake, thinking it will reduce the rashes, even though I was advised not to stop or reduce it.’*

Another participant from Ife reported the following:

*‘I was somehow discouraged from taking the drug, especially when I first started. I had poor appetite. When I forced myself to eat, I would vomit.’*

On the premise that client-provider relationships may influence adherence, questions were asked about the attitude of care providers and how it influenced adherence to ART. The majority of the participants from the two sites described the attitude of providers as very pleasant and quite encouraging. The participants elaborated that counselling by these workers has helped tremendously in ensuring adherence to ART. When asked how adherence could be improved, the PLWHA suggested various ways, including the use of a mobile phone alarm or reminder tones, increasing access to ARV drugs by decentralising the treatment centres and addressing the issues of stigma in the society.

## DISCUSSION

The socio-demographic profile of the participants showed that the majority (70.8%) were in the age group of 15 to 44 years, with a female preponderance. This is consistent with findings of surveys that identified people of this age group as most vulnerable to HIV/AIDS because they belong to the sexually active, reproductive age group.^23, 24^ The findings relating to the high proportion of female PLWHA in this study also support other findings that HIV infection is more common in women than men. Socio-cultural and economic factors that put women at a disadvantage are responsible for this difference.^25^

About half of the participants were currently married while significant proportions were either widowed or unmarried. This distribution of marital status becomes relevant, considering the fact that heterosexual transmission accounts for 80% of HIV infection in developing countries.^24, 25^ A substantial proportion of the subjects were unemployed, while those who were employed were artisans in the low socio-economic class. This was clearly demonstrated during focus group discussions, when a lack of funds for investigations was given as a strong reason for poor adherence to ART. Membership of a support group was reported in a majority of subjects from Ilesa. The PLWHA enjoyed tremendous comradeship in these groups and regular attendance at the fortnightly meetings was associated with good adherence to ART.

The overall adherence level among the study population was 44%. Although the majority of subjects who accessed ARV drugs from LIHOC, Ilesa, had good adherence, the converse was true for subjects who accessed ARV drugs from Ile-Ife. Nevertheless, the findings of this study suggest that the adherence rate among PLWHA to ART in the Ife-Ijesa zone of Osun State is comparable to those in most countries. In developed countries, the rates of patient-reported adherence range from 40% to 70%.^8^ Also, the level of adherence observed in the present study is consistent with findings in Uganda,^26^ South Africa^27^ and Senegal.^28^

The low adherence rate from Ile-Ife may be explained by a number of factors. Firstly, subjects from Ile-Ife had to pay for certain investigations before they can benefit from the free ARV drugs, whereas the subjects from Ilesa only needed to provide transport fare to Lagos where the investigations were done free of charge and they commenced medication subsequently. Another factor that might have contributed to the relatively high adherence rate among participants from Ilesa is the nature and quality of support received from the care institution. Adherence and psychosocial counselling services were offered to PLWHA on ART in LIHOC, Ilesa, while such services were barely available in Ile-Ife at the time of this study. The moral and psychological support received from the group in Ilesa probably cushioned the negative effects of stigma and discrimination of PLWHA by some families and members of the community, and thereby improved adherence. Further studies employing an objective assessment of social support among PLWHA may confirm this conclusion.

Moreover, a significant positive association was obtained between sources of ART and adherence level. This factor remains a strong predictor of adherence to ART on multiple logistic regression analyses. This agrees with the findings of Sheri et al.,^20^ who reported a higher adherence level in private sectors in Botswana than in public health facilities.

Education status had a significant association with adherence level in this study. Also, employment status and monthly income, which are directly dependent on education status, also showed significant association with adherence level. Perception of the cost as affordable or expensive was also significantly related to adherence level. These socio-demographic factors were reported by Nwokike^21^ to correlate well with good adherence to ART. He submitted that if cost is removed as a barrier, adherence is predicted to increase considerably. Our findings further revealed a significant association between frequency of ARV drug use and adherence level. This was stressed during focus group discussion during focus group discussions as responsible for missing some doses of ARV drugs. A simpler regimen of the medication was reported by other studies as promoting adherence to ART.^13, 19^

Other demographic factors such as age, gender and marital status did not have significant associations with adherence level. This is consistent with findings of other authors such as Glifford et al.,^29^ Kleeberger et al.,^13^ and Byakika-Tusiime et al.^26^

Similarly, experience of adverse effects was found not to be a significant correlate of adherence level in this study. This is contrary to the findings of Max and Sherer, association between frequency of ARV drug use and adherence level. This was stressed during focus group discussion as responsible for missing some doses of ARV ^30^ who report that adverse drug reaction had a significant negative effect on adherence to ART. A lack of association in this study may be due to the fact that a significant proportion of PLWHA might have adjusted to the adverse effects of ARV drugs with time. The attitude of care providers also did not correlate with adherence to ART in this study. This negates the findings of other studies^21, 31, 32^ that established a positive influence of the attitude of care providers on adherence to ART. This discrepancy may be connected with the large sample size in those other studies, in sharp contrast with the relatively small sample size in the present study. Nevertheless, focus group discussion participants generally reported a favourable and encouraging attitude on the part of care givers in this study.

The use of self-report medication adherence to assess medication adherence may be a limitation of this study. Although efforts were made to limit recall of medication to the seven days before the interview, it is possible that subjects over- or under-estimated their adherence to ART. This is further worsened by the inability to corroborate patient self-report of adherence with viral loads and CD4 responses because of financial and logistic constraints of frequent laboratory monitoring. However, the use of focus group discussions in this study revealed a number of behavioural factors that might not have been elicited by quantitative instruments.

In conclusion, adherence is an important predictor of viral suppression, reversal of progression to AIDS and death. The maximisation of treatment outcomes with expanding access to ART in resource-limited settings such as Nigeria will require a thorough understanding of the adherence barriers that are unique to resource-poor settings. This therefore implies that further studies are needed to objectively examine the impact of social support on the level of adherence to ART, especially in resource-constrained settings where other support systems are limited. Because the cost of accessing services was a demonstrable barrier to ART adherence, it is recommended that ART centres be decentralised into primary health care units that are closer to people's homes and places of work. The needed facilities and manpower should also be provided in order to make ART more accessible and affordable. Government should make both preliminary investigations for commencement of ART and ART free at all levels of care. This will improve access to ARV drugs and enhance adherence to the medication. There is also an urgent need for an effective and sustainable information, education and communication strategy nationwide to address the problem of HIV stigma and discrimination, which is a proven barrier to care and adherence to therapy. Government and non-governmental agencies are particularly enjoined to make this a priority for lasting HIV/AIDS prevention and control in the country.
